# A 2-Cys peroxiredoxin in response to oxidative stress in the pine wood nematode, *Bursaphelenchus xylophilus*

**DOI:** 10.1038/srep27438

**Published:** 2016-06-07

**Authors:** Zhen Li, Qingwen Zhang, Xuguo Zhou

**Affiliations:** 1 Department of Entomology, China Agricultural University, Beijing, 100193, China; 2 Department of Entomology, University of Kentucky, Lexington, KY, 40546, USA

## Abstract

The pine wood nematode, *Bursaphelenchus xylophilus*, is the causal agent of pine wilt disease that has devastated pine forests in Asia. Parasitic nematodes are known to have evolved antioxidant stress responses that defend against host plant defenses. In this study, the infestation of whitebark pine, *Pinus bungean*, with *B. xylophilus* led to a significant increase in plant hydrogen peroxide (H_2_O_2_) and salicylic acid levels. Correspondingly, the expression of an antioxidative enzyme, 2-Cysteine peroxiredoxin (BxPrx), was elevated in *B. xylophilus* following the H_2_O_2_ treatments. Recombinant BxPrx, a thermal stabile and pH tolerant enzyme, exhibited high level of antioxidant activity against H_2_O_2_, suggesting that it is capable of protecting cells from free radical attacks. Immunohistochemical localization study showed that BxPrx was broadly expressed across different tissues and could be secreted outside the nematode. Finally, the number of BxPrx homologs in both dauer-like and fungi-feeding *B. xylophilus* were comparable based on bioinformatics analysis of existing EST libraries, indicating a potential role of BxPrx in both propagative and dispersal nematodes. These combined results suggest that BxPrx is a key genetic factor facilitating the infestation and distribution of *B. xylophilus* within pine hosts, and consequently the spread of pine wilt disease.

Pinewood nematode, *Bursaphelenchus xylophilus* (Steiner and Buhrer) Nickle, is a pathogenic parasite of the devastating epidemic pine wilt disease in Asia^1^ and Europe^2^, and has caused serious damage to pine forests, especially in Japan^3^ and China^4^. In addition to the economic importance as a notoriously effective plant pathogen, *B. xylophilus* also has drawn attention for its complex ecological traits centered around the tritrophic interactions among insect vectors, plant parasitic nematodes, and pine/fungi hosts^5^. To complete its life cycle, *B. xylophilus* switches from a propagative to a dispersal stage^6^. In the propagative portion of the lifecycle, *B. xylophilus* grow and develop through four juvenile stages and reach adulthood by feeding on the parenchymal cells of the healthy pine trees. This feeding by nematodes leads to the disruption of water transportion and subsequent death of affect tissues in pine trees^7^. When the host plant becomes seriously infected, *B. xylophilus* enters the mycophagous phase wherein nematodes feed on the blue stain fungi colonized in the dying tree until the food sources become limited. The dispersal life cycle starts with the formation of the pre-dauer juveniles that aggregate around the pupal chambers of the pine sawyer beetles, *Monochamus alternatus* Hope. Finally, *M. alternatus* beetles feed on healthy pine wood after emergence, and incidentally brought *B. xylophilus* dauer with them^8^. Subsequently, the dispersal of *M. alternatus* facilitates the spread of beetles and pathogens. The transmission of pine wilt disease would fail unless the three factors, the pine host, parasitic nematode, and insect vector, work in sync^7^.

To ensure successful infestation, *B. xylophilus* needs to break through the pine host defense system. Unlike other plant parasitic nematodes, such as cyst and root-knot nematodes which are sedentary and feed on host plant cells, *B. xylophilus,* a migratory endoparasitic nematode, has to invade and move through the pine host to feed. To counteract host defense, *B. xylophilus* has evolved multiple measurements^9^. On the other hand, host plants activate their defense mechanisms locally and systemically in response to pathogenic attacks^10^ as well as nematode infestations^11^. Reactive oxygen species (ROS), including superoxide anion (O_2_−·), hydrogen peroxide (H_2_O_2_), and hydroxyl radicals (·OH), are considered to be the first line of defense in plants^12^. Reactive oxygen species oxidize DNA, proteins, and lipids, which causes damage to organelles and inhibits cell functions in plant attackers^13^. H_2_O_2_ can cross the plasma membrane and activate signaling molecules to induce defensive genes and enzymes. Specifically, H_2_O_2_ acts as a marker in pathogen recognition, causal trigger for hypersensitive reaction and apoptosis of host plants. Reactive oxygen species, particularly H_2_O_2_, is an important factor for regulation of host-nematode interactions and partly govern the success or failure of disease^14^,^15^. Salicylic acid (SA) is an important signaling molecule involved in systemic resistance by inducing the expression of pathogenesis-related (PR) proteins in plants^16^. A feature of SA signaling is its interaction with ROS in plants^17^. The increase of H_2_O_2_ titer at the site of nematode penetration could induce the accumulation of SA. *Vice versa*, SA could regulate H_2_O_2_ level through manipulating ROS-scavenging systems^18^,^19^. Durner *et al*. propose that H_2_O_2_ might play a role in the activation of PR genes by interaction with SA^10^. Therefore, breaking down ROS defense is not only important to the initial invasion, but also facilitate its ongoing and persistent infestations by weakening the resistant in host plants. Neutralizing ROS is essential for parasitic nematodes to overcome plant defenses^20^. Consequently, the survival of nematodes is directly linked to the antioxidant enzymes^21^.

In many parasitic nematodes, 2-Cysteine peroxiredoxins (Prxs) are overexpressed and involved in H_2_O_2_ detoxification^22^. In animal parasitic nematodes, the secretion of Prxs is considered an evolutionary adaptation to protect nematodes against host H_2_O_2_^23^. *Onchocerca volvulus*, the parasitic nematode for ‘river blindness’, excreted a 2-Cys Prx that localized within the hypodermis and cuticle in the infective larvae^24^. Among plant parasitic nematodes, 2-Cysteine peroxiredoxin (Prx2) has been found on the cuticle surface of infective and post-infective juvenile *Globodera rostochiensis*^25^,^26^, and excreted by *Meloidogyne incognita* when in close contact with plant cells^14^. Furthermore, silencing of Prx2 by RNAi led to the impairment of *M. incognita* development inside the host^14^. These results suggested that Prxs, especially Prx2, may play a critical role in plant-nematode interactions by counteracting plant defensive H_2_O_2_^27^.

Previously, a 2-Cys *Prx* gene from *B. xylophilus, BxPrx*, was cloned and its expression profile was analyzed^28^. Using an integrative approach, we comprehensively characterized *BxPrx* to gain a better understanding of its ecological and biological functions in the co-evolution of pine-nematode arm race. The combined results demonstrated that *BxPrx* plays a key role in the dispersal of *B. xylophilus*, acting as a scavenger for host-derived ROS attack in the propagative cycle, and may protect against its own metabolic H_2_O_2_ in the dispersal cycle.

## Results

### Nematode, plant defensive compounds and BxPrx

**Figure 1:**
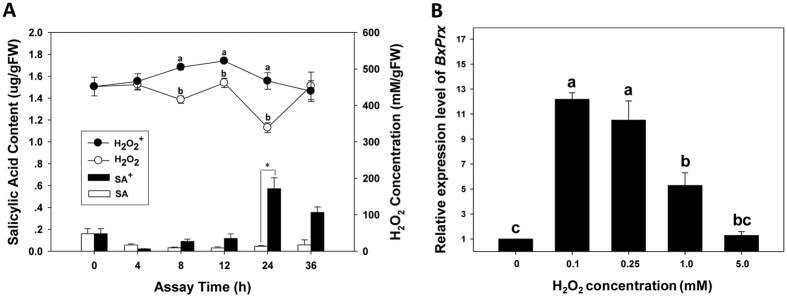
Interactions between pine hosts and nematodes involving BxPrx. (**A**) Comparative analysis of H_2_O_2_ and SA levels between pine seedlings inoculated with *B. xylophilus* (with “+” sign) and control pine inoculated with M9 buffer (without “+” sign). H_2_O_2_ and SA levels were documented at 0, 4, 8, 12, 24, and 36 h post-inoculation. (**B**) After incubated with 0, 0.1, 0.25, 1 and 5 mM H_2_O_2_ for 5 min, *BxPrx* expression was recorded.

The roles of plant secondary metabolites, including H_2_O_2_ and SA, have been investigated in this study. H_2_O_2_ titer in pine hosts were significantly increased after inoculation with *B. xylophilus* for 8 h, peaked at 12 h post inoculation, and high level of H_2_O_2_ was sustained for about 16 h before it dropped back to the level of control pines (Fig. 1A). Similarly, SA titer in pine was significantly induced after inoculation with *B. xylophilus*, and it peaked at 24 h post-inoculation (Fig. 1A).

The stability of four candidate reference genes, including *EF 1α (eukaryotic translation elongation factor 1α*, accession number: GU130132), *α-tubulin* (GU130151), *β-tubulin* (AB500150), and *β-actin* (EU100952), under an array of hydrogen peroxide treatments were examined by quantitative real-time PCR (qRT-PCR) analysis. GeNorm analysis demonstrated *β-actin* (0.400) and *EF 1α* (0.493) had the lowest M-values, suggesting that this pair was the most stably expressed reference genes in *B. xylophilus* under oxidative stress (Table S1)^29^. *BxPrx* expression in *B. xylophilus* was found significantly induced in respond to elevated H_2_O_2_ titer (0.1 ~ 1.0 mM) in the environment, although the expression of *BxPrx* started to decrease when H_2_O_2_ concentration reached 1.0 mM (Fig. 1B).

**Figure 2:**
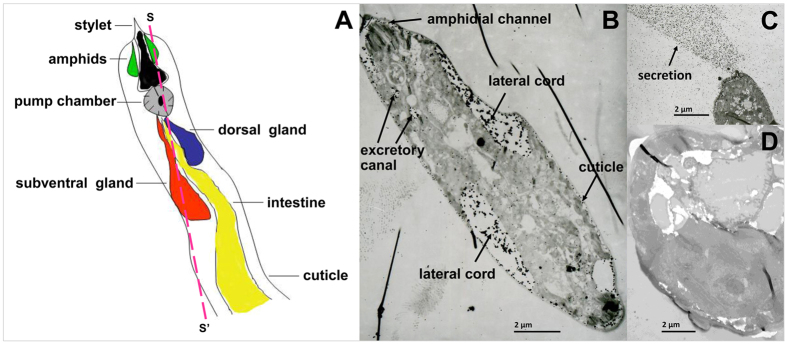
Immunohistochemical localization of BxPrx in *B. xylophilus*. (**A**) Schematic drawing of the anterior section of *B. xylophilus*. It is modified from Vanholme *et al*.^60^, and the dash line indicates the sample section in *B. xylophilus*; (**B**) BxPrx expression was detected under cuticle, in amphid, dorsal and subventral gland, and under cuticle; (**C**) BxPrx was secreted outside the nematode through stylet; (**D**) Preimmune serum showed no detectable binding on the nematode samples.

Immunohistochemical localization study showed that BxPrx was abundantly expressed in *B. xylophilus* and predominantly distributed in excretory canal, and the two lateral glands (Fig. 2B). BxPrx also could be secreted outside the nematode through stylet (Fig. 2C). Preimmune serum showed no detectable binding to nematodes (Fig. 2D).

### Antioxidant activity of recombinant BxPrx

**Figure 3:**
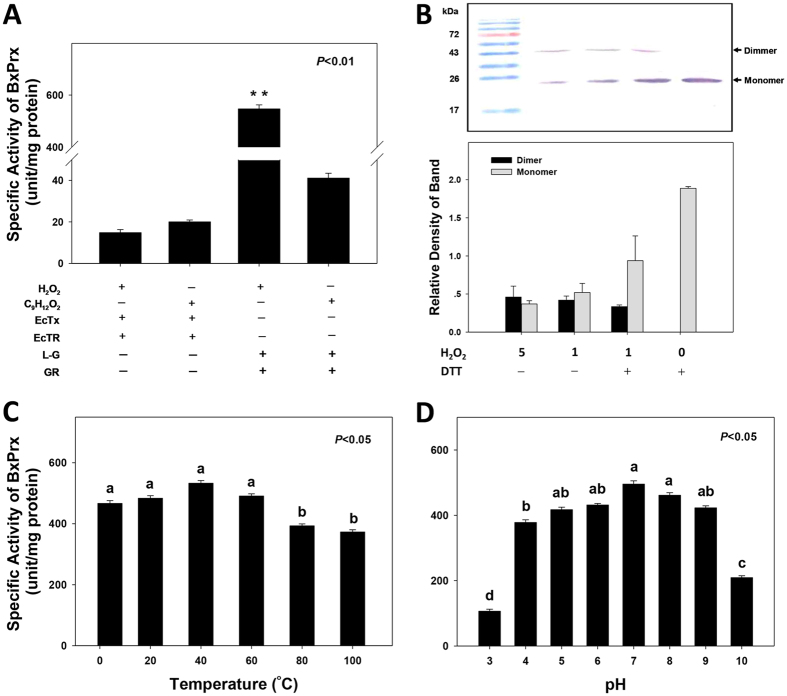
Characterization of BxPrx activity. All assays were done in triplicates, with 1.5 μg of purified recombinant BxPrx in each 500 μl reaction system. (**A**) TPx activity was test with EcTrx and EcTR in the reaction; GPx activity was test with L-G and GR in the reaction; H_2_O_2_ and C_9_H_12_O_2_ were used as substrates separately. (**B**) Immunoblot assay of total protein extract from *B. xylophilus* incubated with 0, 1 mM and 5 mM H_2_O_2_ by polyclonal antiserum against BxPrx. 80 μg protein extract was loaded in each lane separately, and the change from monomer to dimer was detected. Monomer was showed below 26 kDa, and dimer was around 43 kDa. Band density analysis resulted from three replicates. (**C**) GPx activity of BxPrx after incubated at different temperature for 30 min, and the tests were performed in the reaction system of pH 7.0. (**D**) GPx activity of BxPrx under different pH conditions, the tests were performed at room temperature.

Recombinant BxPrx exhibited limited thione peroxidase (TPx) activity in a reaction system containing both thioredoxine (produced from *E. coli*, EcTx) and thione reductase (produced from *E. coli*, EcTR). In contrast, in a reaction system containing L-glutathione and glutathione reductase (GR), BxPrx showed substantial GPx activity (563.8 ± 41.6 units/mg protein) against H_2_O_2_, however, it did not reduce cumene hydroperoxide (C_9_H_12_O_2_) (Fig. 3A). In optimal condition assays, BxPrx exhibited GPx activity between pH 5 and 9, with pH 7and 8 recorded the highest activities, whereas pH 3 and 10, respectively, led to a dramatic reduction of the antioxidant activity (Fig. 3C). BxPrx activity was stable across a wide range of temperature (4–60 °C), however, its antioxidant activity was significantly decreased when temperature reached 80 °C (Fig. 3D).

The conformational change of BxPrx from monomeric to dimeric protein was induced by the addition of H_2_O_2_ (Fig. 3B). The rate of dimerization is positively correlated with H_2_O_2_ concentration. Consequently, BxPrx is a typical 2-Cys Prx with the formation of an inter-molecule dimer to complete the catalytic cycle.

**Figure 4:**
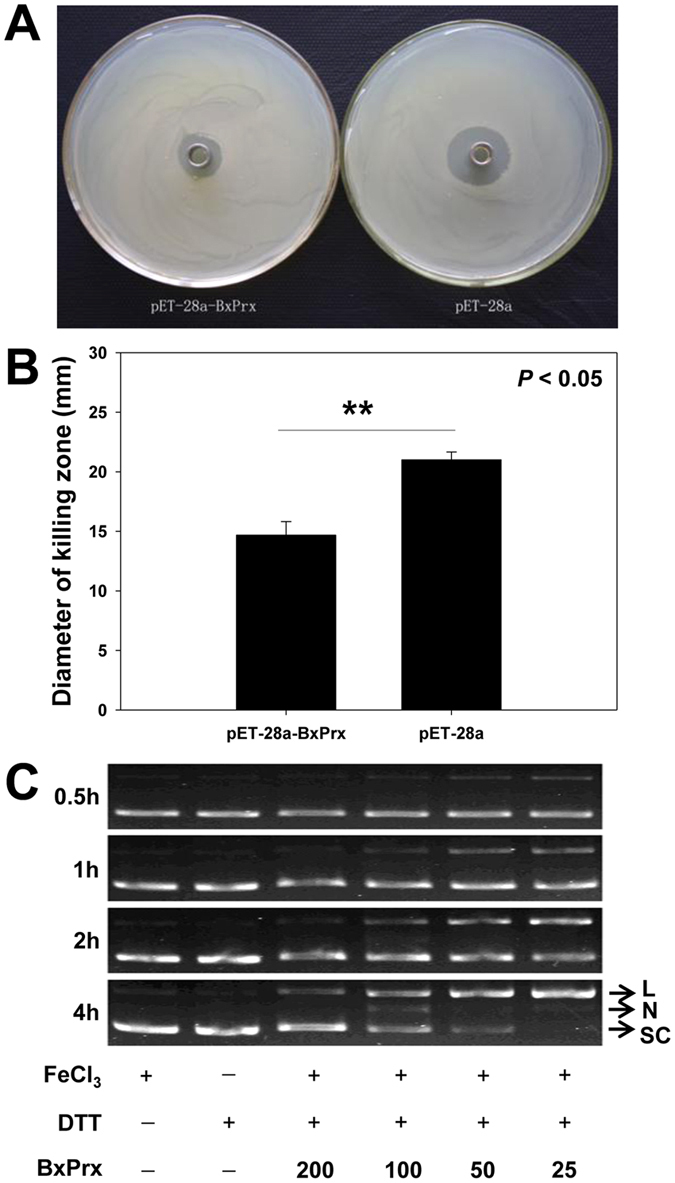
Antioxidant activity of BxPrx *in vitro*. (**A**) Antioxidant activity of *BxPrx* transformants was investigated using a discs assay. (**B**) Difference of susceptibility to H_2_O_2_ between *E. coli* with pET-28a-BxPrx and blank pET-28a transformed were compared by the killing zone diameter. (**C**) DNA protection assay. Free oxidant radical attack was generated due to the presence of Fe^3+^ and DTT. PUC19 plasmid was incubated for 0.5, 1, 2, and 4 h respectively. After attacked by the radical, supercoiled plasmid (SC) was degraded to slower-moving nicking (N), and then slowest linear DNA (L).

Recombinant BxPrx showed significant tolerance to the toxicity of H_2_O_2_ oxidation (30% lower susceptibility than a blank, *P* < *0.05*, Fig. 4A,B). DNA cleavage protection test demonstrated that BxPrx protected plasmid DNA against the free radical attack, and this protection weakened when the amount of BxPrx proteins declined. During a 4 h-observation, supercoiled plasmid DNA was best protected at a BxPrx concentration of 200 μg/μl. The damaged DNAs, such as nicked and slowly moving linear DNAs, became evident when less BxPrx was added into the reaction system. When BxPrx concentration reduced to 25 μg/μl, only the linear DNAs were detected (Fig. 4C).

### Antioxidants among dauer-like and fungi-feeding *B. xylophilus*

Genes encoding antioxidants, including *SOD (superoxide dismutase*), *GPX* and *CAT (catalase*), were extracted from EST libraries generated from dauer-like and fungi-feeding *B. xylophilus*, respectively^30^. In general, fungi-feeding nematodes had a greater number of antioxidants than dauer-like *B. xylophilus*^30^. *BxPrx* homologs, however, showed relatively stable expression between fungi-feeding (Genbank ID: CJ984042, CJ984159, CJ984424, and CJ984735) and dauer-like *B. xylophilus* (Genbank ID: CJ989294, CJ989141 and CJ988426) in comparison to expressions of housekeeping genes, *Act1* and *Hsp70* (Table 1).

## Discussion

### Prx2s in nematodes with different ecological niches

Peroxiredoxin was first detected in the yeast *Saccharomyces cerevisiae*, where its expression was induced by exposure to oxidative stress^31^. In free living bacterivore, *Caenorhabditis elegans,* expression of *CePrx2* was induced by short-term exposure to t-butyl hydroperoxide (t-BOOH)^32^. In an animal parasitic nematode, *Haemonchus contortus*, HcPrx2, exhibited high *Km* toward H_2_O_2_, suggesting that HcPrx2 can protect the nematode against the host immune response^23^. Similarly, in a plant parasitic nematode, *G. rostochiensis*, a 2-cys peroxiredoxin could breakdown H_2_O_2_ and was located on the surface of invasive and post-infective juveniles, suggesting that Prx may play a role in protection against plant defensive responses^25^. MiPrx2, a Prx2 from the other plant parasitic nematode, *M. incognita*, was located in tissues in close proximity with plant cells during parasitism. The development of nematode was impaired after the silencing of *MiPrx2*^14^. *Bursaphelechus xylophilus* has a complex life cycle and unique feeding behavior in comparison to other plant parasitic nematodes which feed primarily on plant tissues for nutrients. It resides two distinctly different habitats: 1) insect vectors for dispersal and foraging, and 2) pine hosts for food, shelter, and proliferation. Moreover, *B. xylophilus* also exploits a wide range of fungi as food source which occupy the dying pine hosts caused by the infestation of *B. xylophilus*^7^. Thus, we hypothesize that genetic factors which suppress or counter the innate immune responses from the pine hosts are essential for the infestation and distribution of *B. xylophilus*. BxPrx, a conserved 2-Cys peroxiredoxin protein with notable antioxidant activity^28^, is likely the genetic factor facilitating *B. xylophilus* to circumvent the host immune response to ensure a successful infestation.

### Host innate immune response to *B. xylophilus* infestation

H_2_O_2_ titers and SA levels were induced in pine seedlings against the infestation of *B. xylophilus*. The induction of SA was lagging behind the release of H_2_O_2_, indicating that ROS was likely to be the first line of defense in pine host innate immune response to *B. xylophilus* infestation. As one of the ROS species, H_2_O_2_ is an early stress signal interacting with a network of signal transduction in plants, including SA pathway to promote defensive responses and systematic acquired resistance (SAR) against pathogens^33^,^34^. Following *B. xylophilus* infestation, elevated H_2_O_2_ level led to the induction of SA pathway and the initiation of SAR in pine hosts. Van Camp *et al*. reported that H_2_O_2_, not SA, might induce the expression of a resistant gene *AoPR-1* in tobacco, indicating that certain defense genes in plant genomes could be activated directly by H_2_O_2_^35^. Researchers also suggested that H_2_O_2_ and SA constitute a self-amplifying system, in which H_2_O_2_ induces SA accumulation and, in turn, SA can increase H_2_O_2_ level^36^. These studies demonstrate that H_2_O_2_ and SA are important plant defensive compounds to deter invading pathogens and to initiate SAR.

### Antioxidant activity of BxPrx

BxPrx has significant antioxidant activity toward H_2_O_2_ in a reaction system containing L-glutathione and GR, but limit activity in a system composed of EcTx and EcTR or on organic oxidants. Some human and animal parasites can use both reaction systems for regeneration, such as *Schistosoma mansoni*^37^ and *Plasmodium falciparum*^38^, although HcPrx could not utilize the glutathione system directly^23^. Prxs in the potato cyst nematode *G. rostochiensis* and plant parasitic root-knot nematode *M. incognita* exhibited notable antioxidant activity in a system that used both trypanothione and thiocyanate as thione donors^14^,^25^. MiPrx2, CePrx2 and HcPrx2 all exhibited strong activities for the reduction of H_2_O_2_ and organic oxidants. Similar to BxPrx, Prx in *G. rostochiensis* was not active against organic oxidants^25^. The different substrate specificity exhibited by these Prxs may be correlated with the habitats occupied by these nematodes. In animals, acquired immunity shows its major capacity in suppressing pathogen infection, however, plants mainly rely on innate immune responses, including ROS and accompanied signal molecules to initiate SAR^39^,^40^. Prxs, with strong antioxidant activity to breakdown H_2_O_2_ and protect DNA from oxidative damage, facilitate *B. xylophilus* infestation by down-regulating the defensive responses in pine hosts.

*Bursaphelenchus xylophilus*, an invasive nematode with a wide range of distribution^41^, shows strong tolerance toward cold and heat stresses^5^,^42^ (11 to 32 °C). In this study, recombinant BxPrx were active against H_2_O_2_ under a broad range of temperature and pH, and protected DNA from oxidative damages. Based on the catalytic mechanisms and the presence of either one or two highly conserved cysteine residues, Prxs are categorized into 1-Cys, typical 2-Cys, and atypical 2-Cys Prxs^43^. The primary structure of BxPrx contains two highly conserved active sites (Cp and Cr). Catalytic reactions of the typical 2-Cys are accomplished through the formation of intermolecular disulfide. In contrast, inner-molecular disulfide is the result of the reaction catalyzed by the atypical 2-Cys Prx^44^,^45^. The conformational change of BxPrx from monomer to inter-molecular dimer was detected after it was oxidized by H_2_O_2_. The rate of dimerization and H_2_O_2_ concentration was positively correlated, indicating that BxPrx is a typical 2-Cys Prx.

### BxPrx in the propagative and dispersal cycle of *B. xylophilus*

The combined results in this study demonstrated that oxidative burst (H_2_O_2_) and secondary metabolites (SA) were inducible when pine hosts were infested with *B. xylophilus*. Following *B. xylophilus* infestation, excessive amount of ROS generated in pine hosts contributes to the development of disease symptoms^46^,^47^. Effective antioxidant activities are an adaptation in pathogens to neutralize host innate defenses to enhance their survivorship and infestation. Previous studies demonstrated that virulent *B. xylophilus* isolates survived better than avirulent counterparts under the oxidative stress^9^,^48^. The bacteria symbiont *Serratia* spp^9^. and elevated *catalase* expression in the intestine of *B. xylophilus*^49^ contribute their tolerance towards the oxidative stress and facilitate the infection of pine wood disease. In this study, *BxPrx* expression was significantly induced by the increase of environmental H_2_O_2_ titer. Recombinant BxPrx showed high levels of antioxidant activity to breakdown H_2_O_2_ and protect DNAs from oxidative radical attacks. *Vice versa*, decreased H_2_O_2_ level also reduce the accumulation of SA and the subsequent host plant innate immune responses. In addition, BxPrx were broadly expressed under cuticle surface where is in close contact with pine hosts, in excretory canals and two lateral glands which are the main secretory organs in nematodes. BxPrx could also be secreted outside the nematode through stylet. These combined results suggest that BxPrx plays a critical role in protecting *B. xylophilus* against pine host-derived H_2_O_2_.

Prx2 is also essential for the development and reproduction of nematodes. Silencing of *CePrx2* in *C. elegans* resulted in a stunted development and a significantly reduced brood size^32^. Similarly, knocking down *BxPrx* significantly reduced the propagation in *B. xylophilus*^50^. Our previous study found that anti-serum of BxPrx could only detect protein samples extracted from virulent *B. xylophilus* but not *B. mucronatus*^28^, suggesting that BxPrx could be used as a molecular marker to identify the virulent *B. xylophilus*^50^. A shorter life cycle and higher proliferation rate are the fundamental differences between avirulent and virulent *B. xylophilus*, therefore, BxPrx could play a role in the pathogenicity of *B. xylophilus*. Different gene clusters were identified between the EST libraries derived from dauer-like and fungi-feeding *B. xylophilus*^30^. Many homologs of antioxidant genes, including *SOD, GPX* and *CAT*, which were involved in longevity in *C. elegans*^32^, were found with notably reduced number in dauer-like EST library compared to fungi feeding *B. xylophilus*^30^. In contrast, *BxPrx* isoforms were similar between dauer-like and fungi-feeding EST libraries. *BxPrx* expression was consistent in the dauer stage, a life stage that is structurally and physiologically adapted for the long-term survival and dispersal without much food sources, suggesting that BxPrx may play an important role against the nematode’s own metabolic ROS for survival during its dispersal cycle.

### Summary

**Figure 5:**
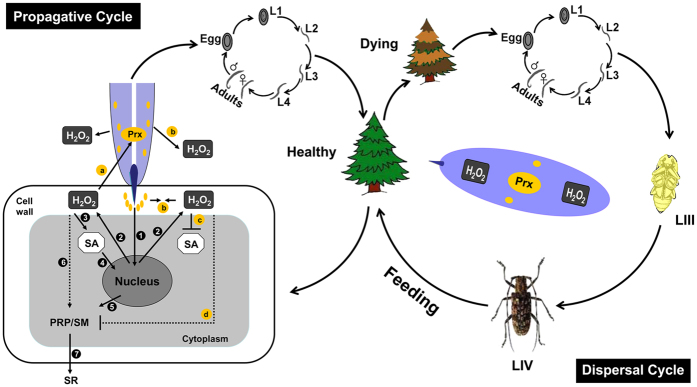
Proposed role of BxPrx in the pine-nematode-pine wilt disease tritrophic interactions. In the propagative cycle, BxPrx protects *B. xylophilus* from the damage of host ROS defense, helps the nematode for a successful infestation into a healthy pine, and then *B. xylophilus* finish the life cycles with egg, four instars of larvae and adults (**1**) The stylet of *B. xylophilus* injected into pine cell for feeding. (**2**) Infestation of *B. xylophilus* could induce H_2_O_2_ defense in pine (**3**) The accumulation of H_2_O_2_ could lead to the increase of SA in host plant. (**4–7**) The accumulation of H_2_O_2_ and SA induced the expression of pathogenesis-related protein (PRP) and signal molecular (SM) which lead to the following systematic resistance (SR) reactions in pines. Correspondingly, (**a**) Expression of BxPrx in *B. xylophilus* could be induced by the H_2_O_2_ from host defense reaction. (**b**) Prx could be secreted to the cuticle surface of nematodes and into host from stylet to neutralize H_2_O_2_ produced by host. (**c,d**) The neutralization of H_2_O_2_ by BxPrx and the following inhibition of the SA accumulation, all would lead to the retard of SR reactions in host. After dramatic reproduction of *B. xylophilus*, host pines become dying, and the dispersal cycle of the nematodes startup. In the dispersal cycle, dauer LIII and LIV nematodes appear and are distributed by the feeding of intermediate insect *M. alternatus*. BxPrx protects *B. xylophilus* against the ROS damage generated from its own metabolism until new infestation.

To summarize our finding, we schematically proposed the multiple functions of BxPrx in the nematode-pine arm race (Fig. 5). During the propagative cycle, the infestation of *B. xylophilus* can induce the production of H_2_O_2_ in pine hosts. Besides direct toxicity to *B. xylophilus*, the accumulation of H_2_O_2_ triggers the elevation of SA. It will also induce the expression of resistance genes and initiate the SAR pathway in pine hosts. In the meantime, however, the elevated H_2_O_2_ level in pine hosts likely induces *BxPrx* expression in nematodes. Synchronized secretion of BxPrx, in turn, reduces H_2_O_2_ level at the micro-environment surrounding *B. xylophilus* in pine hosts and thus protecting nematodes from oxidative damages. This can also reduce the SAR response in pine hosts through the suppression of H_2_O_2_ to a level that the induction of SA pathway is arrested. Therefore, in the propagative cycle, BxPrx plays a critical role in protecting *B. xylophilus* against ROS attack from hosts.

After infestation and proliferation of *B. xylophilus*, hosts show symptoms of wilt and nematodes turn to feed on a variety of fungi occupied in the decaying pine materials. When fungal food sources become scarce, the pre-dauer LIII nematodes congregate around the *M. alternatus* pupae and latch onto the newly emerged adults. The dauer LIV nematodes complete the dispersal cycle through the maturation of the adult pine sawyer beetle on healthy trees. ROS generated from the metabolism can threaten the survivorship of nematodes in the dispersal period. In the dauer stage, antioxidative BxPrx protects nematodes from their own metabolic attacks for a longer life span and a broader distribution during the dispersal cycle.

In conclusion, we suggest that BxPrx is a key genetic factor that facilitates the infestation and distribution of *B. xylophilus* within pine hosts, and consequently the spread of pine wilt disease. With the advent of functional genomics tools, including RNA interference (RNAi)^51^ and transgenic approach^50^, genetic basis governing the transmission and infection of pine wilt disease is a logic choice for future research. A better understanding of how nematodes overcome oxidative stresses generated from their own metabolic processes and from the innate immune responses in pine hosts will provide novel targets for the long-term, sustainable management of this devastating pine disease.

## Materials and Methods

### Biological materials

The *B. xylophilus* isolate JSZJ1-6 (Sampled from Jiangsu, Zhejiang province^1^) was maintained as described previously^52^. Seedlings of 1–2 year-old whitebark pine, *Pinus bungean* (20–40 cm in height and 5–8 cm in diameter), were maintained in a green house with a long-day photoperiod (light/dark 16:8) at 25 °C for 1.5 months until new shoots start to grow.

### Interactions between the pine hosts and nematodes

#### Change of H_2_O_2_ and SA level in pine host responding to B. xylophilus infestation

The innate resistance of *P. bungean* was investigated by an artificial infestation (Fig. S1). Pine seedlings were inoculated with 20,000 *B. xylophilus* per plant^53^ using M9 buffer (KH_2_PO_4_ 30 g/L, K_2_HPO_4_ 60 g/L, NaCl 50 g/L) as a control. Leaves were sampled at 0, 4, 8, 12, 24, and 36 h after inoculation, and snap frozen at −80 °C for the subsequent H_2_O_2_ and SA analysis.

H_2_O_2_ concentration was determined as described previously by Brennan and Frenkel^54^. In brief, 0.5 g of frozen pine needles were ground in 1 ml acetone and centrifuged at 3,000 g for 10 min. After mixing the supernatant with 20% TiCl_4_-HCl solution and ammonium hydroxide, the resultant pellet was harvested and resuspended in 2 M H_2_SO_4_. H_2_O_2_ concentration was measured by A_410_ of the titanium-peroxide complex. Free SA was extracted as described previously^55^. A total of 0.5 g of frozen needles were homogenized in 3 ml of 90% methanol and kept soaking at −20 °C for overnight. After centrifugation at 4 °C, 8,000 rpm, for 30 min, the supernatants were collected and dried in a speed vacuum with heat (~40 °C). The residue was resuspended in 2.5 ml of 5% trichloroacetic acid and sonicated for 10 min. Free SA was then separated from conjugated SA through organic extraction with 2 volumes of ethyl acetate-cyclohexane-isopropanol (50:50:1). The organic phase containing free SA was then dried using a speed vacuum, resuspended in 400 μl of acetonitrile, filtered, and measured by reverse-phase HPLC (HP1100 Agilent, USA). An Agilent-C18 column (5 μm, 250 mm × 4.6 mm), with a detection wavelength of 302 nm and elution condition of V (acetonitrile): V (H_2_O containing 1.5% acetic acid), 3:97 (10 min), 38.8: 61.2 (20 min), 38.8: 61.2 (26 min), was used in the HPLC analysis. The amount of free SA was determined based on the standard curve of a SA reference (Sigma-Aldrich, USA). Paired-samples T test (SPSS17.0, SPSS Inc., Chicago, USA) was used to analyzed changes of H_2_O_2_ and SA levels between pines inoculated with or without *B. xylophilus*.

#### BxPrx expression profile under oxidative stresses

To select the suitable reference gene(s) for the subsequent qRT-PCR analysis, the stability of four housekeeping genes, *EF 1α* (accession number: GU130132), *α-tubulin* (GU130151), *β-tubulin* (AB500150), and *β-actin* (EU100952), under the hydrogen peroxide treatments were evaluated by geNorm^29^. The end point measurement of this conventional statistical software is M-value, the variation of a gene compared to all other candidates. Based on the pair-wise comparison, geNorm automatically eliminates the gene with the highest M-value, and repeats the process until there is only two genes left. The last pair remaining is considered as the optimum pair of reference genes^29^. The PCR primers were listed in Table S2, and the PCR was performed with 40 cycles of 94 °C (30 s), 58 °C (45 s), and 72 °C (30 s).

*Bursaphelenchus xylophilus* of mixed developmental stages were incubated with various concentrations of H_2_O_2_ for 5 min^56^. Total RNA extraction and cDNA synthesis were carried out as described previously^28^. *BxPrx* (GenBank accession number: EU095848) expression profile under oxidative stresses was investigated using qRT-PCR with primers listed in Table S2. qRT-PCR was performed on a 7300 Real-Time PCR System (Applied Biosystems, Foster City USA). The 2^−ΔΔCt^ method was used to calculate the expression level of *BxPrx*. SPSS Statistics 17.0 (SPSS Inc., Chicago, USA) was used for the statistical analyses. Since the data did not fit homoscedasticity, *BxPrx* expression level under oxidative stress was assessed by one-way ANOVA and followed by a Games-Howell test.

### Immunohistochemical localization of BxPrx

*Bursaphelenchus xylophilus* of mixed developmental stages were collected by centrifugation at 2,000 rpm for 1 min, and washed with M9 buffer for 3 times. The resultant nematode samples were fixed, dehydrated, cut to ultrathin sections, hybridized with antiserum, and observed in transmission electron microscope^28^.

### Antioxidant activity of recombinant BxPrx

#### Biochemical characterization of recombinant BxPrx

Recombinant BxPrx protein was expressed in a Novagen’s pET-28a expression vector (Merck KGaA, Darmstadt, Germany) and purified with Ni-NTA agarose (Qiagen, Hamburg Germany)^28^. Optimal conditions for BxPrx activity assay, including substrate specificity, pH tolerance, and temperature range, were determined enzymatically. The capability of recombinant BxPrx on the reduction of H_2_O_2_ and C_9_H_12_O_2_ was measured, respectively, at OD_340_^57^. The reaction system contained 5 mM potassium phosphate (pH 7.0), 1 mM EDTA, 0.1 mM NADPH, and with 16 μM EcTx (Sigma) and 0.23 μM EcTR (Sigma) for the TPx activity assay; or 1 mM L-glutathione (Sigma) and 0.1 unit/ml GR (Sigma) for the GPx activity assay. To determine the optimum pH, BxPrx activity was measured between pH 3 and 10 at room temperature. For optimal temperatures, recombinant BxPrx was incubated for 30 min at a temperature gradient of 4, 20, 40, 60, 80, and 100 °C, respectively, before engaging in a BxPrx activity assay at pH 7. The activities of BxPrx were compared by one-way ANOVA and followed by a Tukey test using SPSS Statistics 17.0 (SPSS Inc., Chicago, USA).

#### Immunoblot analysis

*Bursaphelenchus xylophilus* was treated with H_2_O_2_ and harvested for the assay as previously described by Oláhová^58^. Nematodes were incubated in 0, 1 and 5 mM H_2_O_2_ solution separately for 5 min, and then washed with M9 buffer for 3 times. Nematodes were then harvested in 20% trichloroacetic acid (TCA) before snap-frozen in liquid nitrogen. Thawed pellets were washed with acetone to remove TCA, dried and resuspended in SDS-PAGE loading buffer. The resultant samples were then separated through 12% SDS PAGE and analyzed by Western blotting using an anti-BxPrx serum^35^. Band intensity was analyzed using Quantity One 4.6.2 (Bio-Rad, USA).

#### Antioxidant activity assay

Antioxidant activity of *BxPrx* transformants was assayed following Isermann^32^ with minor modifications. *Escherichia coli* was spread onto the top of a LB (Lysogeny Broth) agar plate (LB medium supplemented with a 2 mm of 0.8% agarose layer below, 1 mM IPTG, and 100 μg/ml ampicillin inside), and then incubated at 37 °C for 3 h. Subsequently, oxford disc (1 cm in diameter) was inserted into the middle of the LB medium and positioned upon the agarose layer. A total of 100 μl of 1 mM H_2_O_2_ was then added onto the disc. The resultant plates were placed at the room temperature for 16 h without moving until all the H_2_O_2_ infiltrated into the LB medium, and then plates were transferred to 37 °C. After 24 h of incubation, the diameters of the killing zones were measured. The viability of *E. coli* transformats with or without BxPrx was compared using a paired-samples T test (SPSS17. 0, SPSS Inc., Chicago, USA).

#### DNA cleavage protection assay

According to Stacy^59^, various concentrations of recombinant BxPrx was first incubated in a reaction mixture containing 250 mM Hepes, 5 mM EDTA, 100 mM DTT, and 600 μM FeCl_3_, at 37 °C for 30 min, and then 250 ng of pUC19 plasmid was introduced into the reaction system (Tiangen, Beijing, China). The status of pUC19 with different incubation conditions was resolved by agarose gel electrophoresis (1%).

#### Transcripts abundance of antioxidant enzymes in B. xylophilus

Transcripts abundance of antioxidant enzymes was analyzed by searching and comparing the number of homologs between EST libraries generated from dauer-like and fungi feeding *B. xylophilus*. The sequence information of the two EST libraries was downloaded from NCBI database, and the retrieval of the homologs was conducted using software BioEdit (Ibis Biosciences, Carlsbad, CA).

## Additional Information

**How to cite this article**: Li, Z. *et al*. A 2-Cys peroxiredoxin in response to oxidative stress in the pine wood nematode, *Bursaphelenchus xylophilus. Sci. Rep.* **6**, 27438; doi: 10.1038/srep27438 (2016).

## Supplementary Material

Supplementary Information

## Figures and Tables

**Table 1:** Antioxidative transcripts extracted from fungi-feeding and dauer-like *B. xylophilus* EST libraries.

	**Transcript**^a^	**Description**	**Number of ESTs**	
			**Fungi-feeding**	**Dauer-like**
Antioxidant	BXC00823	SOD-1	1	0
	BXC04177	SOD-3	1	0
	BXC07336	SOD-3	0	1
	BXC03446	SOD-4	1	0
	BXC03943	GPX	2	0
	BXC00081	GPX	2	3
	BXC01784	GPX	1	0
	BXC02367	CAT-1	2	0
	CJ984042	Prx2	1	0
	CJ984159	Prx2	1	0
	CJ984424	Prx2	1	0
	CJ984735	Prx2	1	0
	CJ989294	Prx2	0	1
	CJ989141	Prx2	0	1
	CJ988426	Prx2	0	1
Housekeeping gene	CJ984848	Actin1	1	
	CJ984265	Actin1	1	
	CJ984341	Actin1	1	
	CJ984728	Actin1	1	
	CJ985146	Actin1	1	
	CJ984832	Actin1	1	
	CJ984026	Actin1	1	
	CJ985003	Actin1	1	
	CJ985085	Actin1	1	
	CJ990129	Actin1		1
	CJ988716	Actin1		1
	CJ990734	Actin1		1
	CJ989041	Actin1		1
	CJ988766	Actin1		1
	CJ990669	Actin1		1
	CJ989163	Actin1		1
	CJ984710	Hsp70	1	
	CJ984856	Hsp70	1	
	CJ989482	Hsp70		1
	CJ988689	Hsp70		1

^a^E-value < E^−10^.

## References

[b1] Cheng X., Cheng F., Xu R. & Xie B. Genetic variation in the invasive process of *Bursaphelenchus xylophilus* (Aphelenchida: Aphelenchoididae) and its possible spread routes in China. Heredity 100, 356–365 (2008). 18091770 10.1038/sj.hdy.6801082

[b2] Dwinell L. D. The pinewood nematode: regulation and mitigation. Annu Rev Phytopathol 35, 153–166 (1997). 15012519 10.1146/annurev.phyto.35.1.153

[b3] Sathyapala S. Pest risk analysis biosecurity risk to New Zealand of pinewood Nematode (*Bursaphelenchus xylophilus*). Ministry of Agriculture and Forestry (2004).

[b4] Shi J., Luo Y., Wu H., Wang L. & Wang G. Z. Traits of Masson pine affecting attack of pine wood nematode. J Integr Plant Biol 49, 1763–1771 (2007).

[b5] Futai K. Pine wood nematode. Bursaphelenchus xylophilus. Annu Rev Phytopathol 51, 61–83 (2013). 23663004 10.1146/annurev-phyto-081211-172910

[b6] Huang Q. . MicroRNA discovery and analysis of pinewood nematode *Bursaphelenchus xylophilus* by deep sequencing. PLoS ONE 5, e13271(2010). 20967259 10.1371/journal.pone.0013271 PMC2953492

[b7] Jones J. T., Moens M., Mota M., Li H. & Kikuchi T. *Bursaphelenchus xylophilus*: opportunities in comparative genomics and molecular host-parasite interactions. Mol Plant Pathol 9, 357–368 (2008). 18705876 10.1111/j.1364-3703.2007.00461.x PMC6640334

[b8] Aikawa T., Kikuchi T. & Kosaka H. Population structure of *Bursaphelenchus xylophilus* within single *Pinus thunbergii* trees inoculated with two nematode isolates. Forest Pathol 36, 1–13 (2006).

[b9] Vicente C. S. L., Ikuyo Y., Mota M. & Hasegawa K. Pinewood nematode-associated bacteria contribute to oxidative stress resistance of *Bursaphelenchus xylophilus*. BMC Microbiol 13, 299 (2013). 24365493 10.1186/1471-2180-13-299 PMC3880045

[b10] Durner J., Shah J. & Klessig D. F. Salicylic acid and disease resistance in plants. Trends Plant Sci 2, 266–274(1997).

[b11] Kim Y. H., Huang F. & Riggs R. D. Resistance of soybean to *Heterodera glycines* and isozyme patterns of peroxidase of soybean roots. *Korean* J Plant Pathol 6, 285–288 (1990).

[b12] Lamb C. & Dixon R. A. The oxidative burst in plant disease resistance. Annu Rev Phytopathol 48, 251–275 (1997). 10.1146/annurev.arplant.48.1.251 15012264

[b13] Baker C. J. & Orlandi E. W. Active oxygen in plant pathogenesis. Annu Rev Phytopathol 33, 299–321 (1995). 18999963 10.1146/annurev.py.33.090195.001503

[b14] Dubreuil G. . Peroxiredoxins from the plant parasitic root-knot nematode, *Meloidogyne incognita*, are required for successful development within the host. Int J Parasitol 41, 385–396 (2011). 21145323 10.1016/j.ijpara.2010.10.008

[b15] Levine A., Tenhaken R., Dixon R. & Lamb C. H_2_O_2_ from the oxidative burst orchestrates the plant hypersensitive disease resistance response. Cell 79, 583–593 (1994). 7954825 10.1016/0092-8674(94)90544-4

[b16] Gaffney T. . Requirement of salicylic acid for the induction of systemic acquired resistance. Science 261, 754–756 (1993). 17757215 10.1126/science.261.5122.754

[b17] Nie S., Yue H., Zhou J. & Xing D. Mitochondrial-derived reactive oxygen species play a vital role in the salicylic acid signaling pathway in *Arabidopsis thaliana*. PLoS ONE 10, e0119853 (2015). 25811367 10.1371/journal.pone.0119853 PMC4374720

[b18] Dat J. F., Foyer C. H. & Scott I. M. Changes in salicylic acid and antioxidants during induced thermotolerance in mustard seedlings. Plant Physiol 118, 1455–1461(1998). 9847121 10.1104/pp.118.4.1455 PMC34763

[b19] Gechev T. S., Breusegem F. V., Stone J. M., Denev I. & Loloi C. Reactive oxygen species as signals that modulate plant stress responses and programmed cell death. Bioassays 28, 1091–1101 (2006). 10.1002/bies.20493 17041898

[b20] Callahan H. L., Crouch R. K. & James E. R. Helminth anti-oxidant enzymes: a protective mechanism against host oxidants? Parasitol Today 4, 218–225 (1988). 15463102 10.1016/0169-4758(88)90162-7

[b21] Henkle-Dührsen K. & Kampköter A. Antioxidant enzyme families in parasitic nematodes. Mol Biochem Parasitol 114, 129–142 (2001). 11378193 10.1016/s0166-6851(01)00252-3

[b22] McGonigle S., Dalton J. P. & James E. R. Peroxiredoxins: a new antioxidant family. Parasitol Today 14, 139–145 (1998). 17040731 10.1016/s0169-4758(97)01211-8

[b23] Hudson A. L., Sotirchos I. M. & Davey M. W. The activity and hydrogen peroxide sensitivity of the peroxiredoxins from the parasitic nematode *Haemonchus contortus*. Mol Biochem Parasitol 176, 17–24 (2011). 21075149 10.1016/j.molbiopara.2010.11.006

[b24] Zipfel P. F., Schrum S., Bialonski A. & Büttner D. W. The peroxidoxin 2 protein of the human parasite *Onchocerca volvulus*: recombinant expression, immunolocalization, and demonstration of homologous molecules in other species. Parasitol Res 84, 623–631 (1998). 9747934 10.1007/s004360050461

[b25] Robertson L., Robertson W. M., Sobczak M., Helder J. & Tetaud E. Cloning, expression and functional characterisation of a peroxiredoxin from the potato cyst nematode *Globodera rostochiensis*. Mol Biochem Parasitol 111, 41–49 (2000). 11087915 10.1016/s0166-6851(00)00295-4

[b26] Williamson V. M. & Gleason C. A. Plant-nematode interactions. Curr Opin Plant Biol 6, 327–333 (2003). 12873526 10.1016/s1369-5266(03)00059-1

[b27] Chandrashekar R. . Removal of hydrogen peroxide by a 1-cysteine peroxiredoxin enzyme of the filarial parasite *Dirofilaria immitis*. Parasitol Res 86, 200–206 (2000). 10726990 10.1007/s004360050032

[b28] Li Z. . Cloning and characterization of a 2-Cys peroxiredoxin in the pine wood nematode, *Bursaphelenchus xylophilus*, a putative genetic factor facilitating the infestation. Int J Biol Sci 7, 823–836 (2011). 21814479 10.7150/ijbs.7.823 PMC3149278

[b29] Vandesompele J. . Accuracy normalization of real-time quantitative RT-PCR data by geometric averaging of multiple internal control genes. Genome Biol 3, research0034.1–research0034 11, (2002). 12184808 10.1186/gb-2002-3-7-research0034 PMC126239

[b30] Kikuchi T. . Expressed sequence tag (EST) analysis of the pine wood nematode *Bursaphelenchus xylophilus* and *B. mucronatus*. Mol Biochem Parasitol 155, 9–17 (2007). 17560668 10.1016/j.molbiopara.2007.05.002

[b31] Kim I. H., Kim K. & Rhee S. G. Induction of an antixoidant protein of *Saccharomyces cerevisiae* by O_2_, Fe^3+^, or 2-mercaptoethanol. Proc Natl Acad Sci USA 86, 6018–6022 (1989). 2668950 10.1073/pnas.86.16.6018 PMC297766

[b32] Isermann K., Liebau E., Roeder T. & Bruchhaus I. A peroxiredoxin specifically expressed in two types of pharyngeal neurons is required for normal growth and egg production in *Caenorhabditis elegans*. J Mol Biol 338, 745–755 (2004). 15099742 10.1016/j.jmb.2004.03.021

[b33] Wu G. . Activation of host defense mechanisms by elevated production of H_2_O_2_ in transgenic plants. Plant Physiol 115, 427–435 (1997). 12223817 10.1104/pp.115.2.427 PMC158500

[b34] Chen Z., Silva H. & Klessig D. F. Active oxygen species in the induction of plant systemic acquired resistance by salicylic acid. Science 162, 1883–1886 (1993). 8266079 10.1126/science.8266079

[b35] Van Camp W., Van Montagu M. & Inzé D. H_2_O_2_ and NO: redox signals in disease resistance. Trends Plant Sci 3, 330–334 (1998).

[b36] Thomma B. P. . Separate jasmonated-dependent and salicylic-dependent defense response pathways in *Arabidopsis* are essential for resistance to distinct microbial pathogens. Proc Natl Acad Sci USA 95, 15107–15111(1998). 9844023 10.1073/pnas.95.25.15107 PMC24583

[b37] Sayed A. A. & Williams D. L. Biochemical characterization of 2-Cys peroxiredoxins from *Schistosoma mansoni*. J Biol Chem 279, 26159–26166 (2004). 15075328 10.1074/jbc.M401748200

[b38] Sztajer H. . The putative glutathione peroxidase gene of *Plasmodium falciparum* codes for a thioredoxin peroxidase. J Biol Chem 276, 7397–403 (2001). 11087748 10.1074/jbc.M008631200

[b39] Nürnberger T., Brunner F., Kemmerling B. & Piater L. Innate immunity in plants and animals: striking similarities and obvious differences. Immunol Rev 198, 249–266 (2004). 15199967 10.1111/j.0105-2896.2004.0119.x

[b40] Trewavas A. J. & Malhó R. Signal perception and transduction: the origin of the phenotype. Plant Cell 9, 1181–1195 (1997). 12237382 10.1105/tpc.9.7.1181 PMC156990

[b41] Kang J. S., Koh Y. H., Moon Y. S. & Lee S. H. Molecular properties of a venom allergen-like protein suggest a parasitic function in the pinewood nematode *Bursaphelenchus xylophilus*. Int J Parasitol 42, 63–70 (2012). 22142561 10.1016/j.ijpara.2011.10.006

[b42] Xie B. . Mechanisms of invasive population establishment and spread of pinewood nematodes in China. Sci China Ser C: Life Sci 52, 587–594 (2009). 19557337 10.1007/s11427-009-0071-y

[b43] Chae H. Z., Chung S. J. & Rhee S. G. Thioredoxin-dependent peroxide reductase from yeast. J Biol Chem 269, 27670–27678 (1994). 7961686

[b44] Wood Z. S., Schröder E., Robbin H. J. & Poole L. B. Structure, mechanism and regulation of peroxiredoxins. Trends Biochem Sci 28, 32–40 (2003). 12517450 10.1016/s0968-0004(02)00003-8

[b45] Jönsson T. J., Johnson L. C. & Lowther W. T. Structure of the sulphiredoxin-peroxiredoxin complex reveals an essential repair embrace. Nature 451, 98–101 (2008). 18172504 10.1038/nature06415 PMC2646140

[b46] Myers R. F. Pathogenesis in pine wilt caused by pinewood nematode *Bursaphelenchus xylophilus*. J Nematol 20, 236–244 (1988). 19290207 PMC2618809

[b47] Iwahori H. & Futai K. Lipid peroxidation and ion exudation of pine callus tissues inoculated with pinewood nematodes. Jpn J Nematol 23, 79–89 (1993).

[b48] Vicente C. S. L., Ikuyo Y., Shinya R., Mota M. & Hasegawa K. Catalases induction in high virulence pinewood nematode *Bursaphelenchus xylophilus* under hydrogen peroxide-induced stress. PLoS ONE 10, e0123839. (2015). 25894519 10.1371/journal.pone.0123839 PMC4404050

[b49] Shinya R. . Secretome analysis of the pine wood nematode *Bursaphelenchus xylophilus* reveals the tangled roots of parasitism and its potential for molecular mimicry. PLoS ONE 8, e67377 (2013). 23805310 10.1371/journal.pone.0067377 PMC3689755

[b50] Fu H., Ren J., Huang L., Li H. & Ye J. Screening and functional analysis of the peroxiredoxin specifically expressed in *Bursaphelenchus xylophilus*-the causative agent of pine wilt disease. Int J Mol Sci 15, 10215–10232 (2014). 24918285 10.3390/ijms150610215 PMC4100149

[b51] Park J. E. . The efficiency of RNA interference in *Bursaphelenchus xylophilus*. Mol Cells 31, 81–86 (2008). 18525237

[b52] Kikuchi T., Jones J. T., Aikawa T., Kosaka H. & Ogura N. A family of glycosyl hydrolase family 45 cellulases from the pine wood nematode *Bursaphelenchus xylophilus*. FEBS Lett 572, 201–205 (2004). 15304348 10.1016/j.febslet.2004.07.039

[b53] Bolla R. I., Fitzsimmons K. & Winter R. E. K. Carbohydrate concentration in pine as affected by inoculation with *Bursaphelenchus xylophilus*. J nematol 19, 51–57 (1987). 19290106 PMC2618607

[b54] Brennan T. & Frenkel C. Involvement of peroxide in the regulation of senescence in pear. Plant Physiol 59, 411–416 (1977). 16659863 10.1104/pp.59.3.411 PMC542414

[b55] Bowling S. A. . A mutation in Arabidopsis that leads to constitutive expression of systemic acquired resistance. Plant Cell 6, 1845–1857 (1994). 7866028 10.1105/tpc.6.12.1845 PMC160566

[b56] Upadhyaya H., Khan M. H. & Panda S. K. Hydrogen peroxide induces oxidative stress in detached leaves of *Oryza sativa* L. *General Appl* Plant Physiol 33, 83–95 (2007).

[b57] Kwatia M. A., Botkin D. J. & Williams D. L. Molecular and enzymatic characterization of *Schistosoma mansoni* thioredoxin peroxidase. J Parasitol 86, 908–915 (2000). 11128509 10.1645/0022-3395(2000)086[0908:MAECOS]2.0.CO;2

[b58] Oláhová M. . A redox-sensitive peroxiredoxin that is important for longevity has tissue-and stress-specific roles in stress resistance. Proc Natl Acad Sci USA 105, 19839–19844 (2008). 19064914 10.1073/pnas.0805507105 PMC2604961

[b59] Stacy R. A. P., Munthe E., Steinum T., Sharma B. & Aalen R. B. A peroxiredoxin antioxidant is encoded by a dormancy-related gene, *Per1*, expressed during late development in the aleurone and embryo of barley grains. Plant Mol Biol 31, 1205–1216 (1996). 8914536 10.1007/BF00040837

[b60] Vanholme B. . Secretions of plant-parasitic nematode: a molecular update. Gene 332, 13–27 (2004). 15145050 10.1016/j.gene.2004.02.024

