# Supplementary material for: A 2-Cys peroxiredoxin in response to oxidative stress in the pine wood nematode, Bursaphelenchus xylophilus
Source: Sci Rep. 2016 Jun 7;6:27438. doi: 10.1038/srep27438 (PMC4895224; doi:10.1038/srep27438)
Supplement: Supplementary Information [file srep27438-s1.doc]

**Supplementary Materials**

**A 2-cys peroxiredoxin in response to oxidative stress in the pine wood nematode, *Bursaphelenchus xylophilus***

Zhen Li ^1,2^, Qingwen Zhang ^1^,*, Xuguo Zhou ^2^,*

^1^ Department of Entomology, China Agricultural University, Beijing, 100193, China

^2^ Department of Entomology, University of Kentucky, Lexington, KY, 40546, USA

----------------------------------------------------------------------------------------------------------------

*** Corresponding Authors:

Dr. Qingwen Zhang

Department of Entomology

China Agricultural University

No.2 Yuanmingyuan West Road

Beijing 100193, China

Email: zhangqingwen@263.net

Dr. Xuguo "Joe" Zhou

Department of Entomology

University of Kentucky

S-225 Agricultural Science Center North

Lexington, KY 40546-0091

Phone: 859-257-3125

Email: [xuguozhou@uky.edu](mailto:xuguozhou@uky.edu)


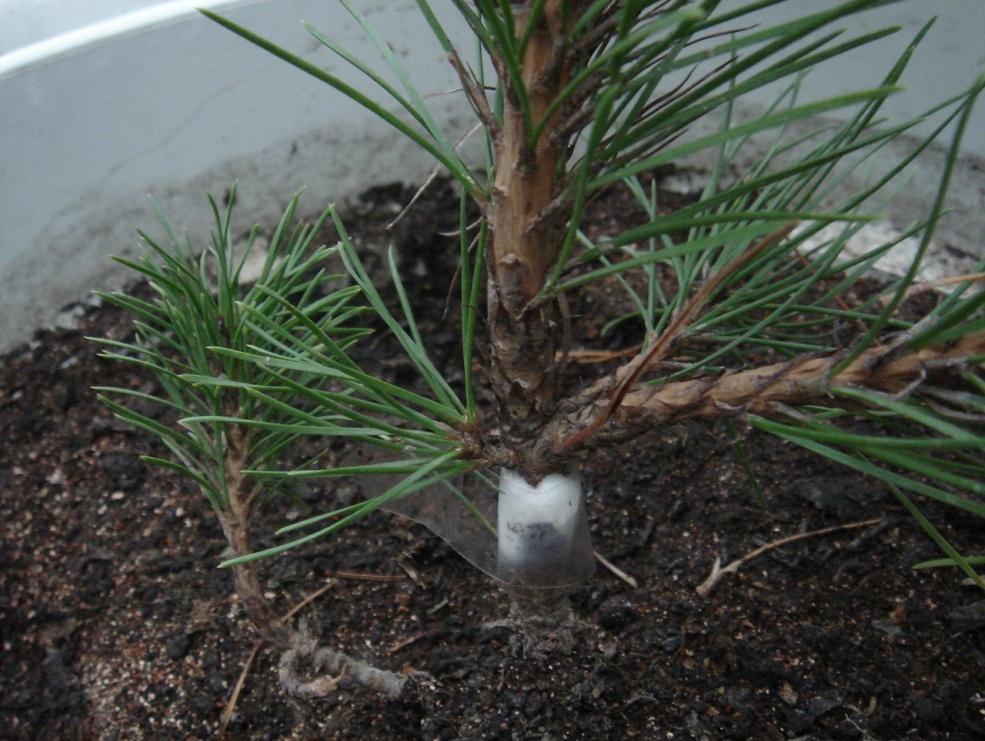


**Figure S1. Inoculation of *B. xylophilus* into a pine host.** The bottom stem of *Pinus bungean* seedling (20-40 cm in height and 5-8 cm in diameter) was gently incised with sterilized scalpel at the 1.5-2 cm below the branch point, and resulted in a wound with a depth about 2/5 of the stem diameter. A small sterilized cotton ball was inserted into the incision, and about 20, 000 nematodes were injected into the cotton ball with pipette. The wound was finally fixed with scotch tape and the seedlings with inoculation were maintained under a photoperiod of light/dark 16:8h at 25ºC.

**Table S1: Stability analysis of reference genes in *B. xylophilus* under oxidative stress**

| **H_2_O_2_ Conc^1^** | ***EF 1α*** | ***α-tubulin*** | ***β-tubulin*** | ***β-actin*** |
| --- | --- | --- | --- | --- |
|  |  |  |  |  |
| 0.00 | 1.14E+01 | 8.12E+00 | 7.43E+00 | 8.26E+00 |
| 0.10 | 2.73E+00 | 1.43E+00 | 3.49E+00 | 2.70E+00 |
| 0.25 | 1.00E+00 | 1.00E+00 | 2.10E+00 | 1.39E+00 |
| 1.00 | 1.84E+00 | 1.54E+00 | 1.81E+00 | 1.76E+00 |
| 5.00 | 1.50E+00 | 1.27E+00 | 1.00E+00 | 1.00E+00 |
| **M-value** | **0.493** | **0.519** | **0.578** | **0.400** |

"^1^": H_2_O_2_ concentration (mM)

**Table S2**: Primer Information

| **Primer ID** | **Primer Sequence (5′-3′)** |
| --- | --- |
| β-actinF | TTGGCTGGCCGTGACTTGAC |
| β-actinR | GCGGTGGCCATCTCCTGTTC |
| EF1αF | TGTTGCTCGTTGGTACAGAA |
| EF1αR | CGCTCTTGCTTGATTCTCTC |
| α-tubulinF | ACAACTCCGTGTTGACCACT |
| α-tubulinR | GCTCCATCGAATCTGAGAGA |
| β-tubulinF | ACTACCTGTCTCCGATTCC |
| β-tubulinR | GCCATCATATTCTTGGCATC |
| actinF | CTTCTTCCCTCGAGAAGTCC |
| actinR | TTGTAGGTGGTCTCGTGGAT |
| BxPrxF | TCGGCGTTGAGGTATTGGC |
| BxPrxR | GTAGTCGCGTGAGATCTTGTGG |
